# Early Substance Use as a Risk Marker in Adolescents: Indicated Prevention in Public Mental Health

**DOI:** 10.1192/j.eurpsy.2026.11142

**Published:** 2026-07-31

**Authors:** L. López Gómez-Miguel, A. Gutiérrez Fernández de Velasco, M. I. Montañés Rodríguez, A. Ladera de la Oliva, E. García Martín de la Fuente, L. M. F. Oliva, R. C. Ramos, M. D. R. D. F. Rodríguez, L. P. Ayuso Morales, M. Andrade Arango

**Affiliations:** 1 Programa de Transición y Primeros Consumos, Hospital Universitario de Toledo, Toledo, Spain; 2 Universidad del Rosario, Bogotá, Colombia

## Abstract

**Introduction:**

Adolescent substance use is recognized as an early marker of vulnerability to severe mental illness, regardless of frequency or intensity. Detecting and addressing initial consumption provides a gateway for cost-effective preventive programs, avoiding the limitations of broad population screening.

**Objectives:**

- To assess substance use as an entry point to early intervention.
- To describe the Toledo program’s experience with adolescents presenting early substance use.
- To analyze the role of brief group psychotherapy in improving outcomes.

**Methods:**

Cohort of adolescents with cannabis, alcohol, polysubstance use, or behavioral addictions (mobile phones, video games, internet). Weekly brief group interventions based on Yalom’s *Here and Now* and Badaracco’s *Healthy Virtuality*, integrated with individual and family sessions.

**Results:**

- Cannabis was the primary reason for referral.
- Growing demand related to behavioral addictions (e.g., smartphones, gaming).
- Over 70% had received prior, often fragmented, treatments.
- Groups facilitated symbolization processes and improved adherence in resistant populations.
- High prevalence of complex trauma and homo/heterotypic relapses (>70%).

**Conclusions:**

Early substance use is a strong clinical and epidemiological marker for indicated prevention. Integrating group-based brief psychotherapies enables effective intervention, enhances adherence, and fosters early detection of psychosis, reducing long-term risk of chronic trajectories.

**Disclosure of Interest:**

None Declared

